# Orthobiologics in delayed union and non-union of adult long bones fractures: A systematic review

**DOI:** 10.1016/j.bonr.2024.101760

**Published:** 2024-04-06

**Authors:** Lorenzo Impieri, Andrea Pezzi, Henrique Hadad, Giuseppe M. Peretti, Laura Mangiavini, Nicolò Rossi

**Affiliations:** a Residency Program in Orthopedics and Traumatology, University of Milan, Milan, Italy; b Department of Diagnosis and Surgery, São Paulo State University (UNESP), Araçatuba School of Dentistry, São Paulo, Brazil; c Department of Biomedical Sciences for Health, University of Milan, Milan, Italy; d IRCCS Ospedale Galeazzi Sant'Ambrogio, Milan, Italy

**Keywords:** Orthobiologics, PRP, Pseudoartrosis, Non-union, Bone healing, Fractures, Delayed unions

## Abstract

**Background:**

Fracture healing poses a significant challenge in orthopedics. Successful regeneration of bone is provided by mechanical stability and a favorable biological microenvironment. This systematic review aims to explore the clinical application of orthobiologics in treating aseptic delayed union and non-union of long bones in adults.

**Methods:**

A systematic review was conducted following the Preferred Reporting Items for Systematic Review and Meta-Analyses (PRISMA) guidelines. Three databases were explored, with no date restrictions, using keywords related to orthobiologics and delayed union and non-union. Eligible studies included human clinical studies in English, with available full texts, examining orthobiologics such as platelet-rich plasma (PRP), mesenchymal stem cells (MSCs), and bone morphogenetic protein (BMPs) for treating aseptic delayed unions and non-unions in adults. Animal studies, in vitro research, and studies on non-unions due to congenital defects, tumors or infections were excluded.

**Results:**

The initial search identified 9417 studies, with 20 ultimately included in the review. These studies involved 493 patients affected by non-union and 256 patients affected by delayed union, with an average age respectively of 40.62 years and 41.7 years. The mean follow-up period was 15.55 months for non-unions and 8.07 months for delayed unions. PRP was the most used orthobiologic, and outcomes were evaluated through time to union, functional scores, and clinical examinations. The results indicated that orthobiologics, especially PRP, tended to yield better outcomes compared to surgical procedures without biological factors.

**Conclusion:**

This systematic review suggests that orthobiologics, such as PRP, BMPs, and MSCs, can be effective and safe in the management of delayed union and non-union fractures. These biological treatments have the potential to improve union rates, reduce healing times, and enhance functional outcomes in patients with non-union fractures. Further research is essential to refine treatment protocols and determine the most suitable orthobiologic for specific patient populations and fracture types.

## 1 Introduction

Long-bone delayed union and non-union are among the most devastating complications of traumatic fractures (Kanakaris and Giannoudis, 2007). Fracture non-union or pseudoarthrosis is the inability to achieve bone healing and union within six months since the injury, with no signs of healing for three consecutive months, and delayed union is the absence of clear radiographic signs of bone consolidation between 4 and 6 months after the injury (Fayaz et al., 2011). According to the Centers for Disease Control and Prevention (CDC), over 6 million fractures occur annually in the USA, substantially contributing to morbidity and disability (CDC, 2021). Roughly 5–10 % of patients with fractures encounter problematic fracture healing and non-union (Nauth et al., 2018). It is a persistent, painful condition that significantly impacts the patient's quality of life with substantial medical expenses and delays the resumption of work, creating a socioeconomic burden (Tay et al., 2014; Hak et al., 2014).

The unique intrinsic bone healing ability is a complex biological and biomechanical process. After trauma and under appropriate conditions, bone healing is characterized by three phases: the inflammatory phase, the repair phase, and the remodeling phase. A non-union can be caused by congenital defects, oncological resections, necrosis, osteomyelitis, and high-energy traumas or impairments during the typical stages of bone repair, impacting the fracture's mechanical and/or biological characteristics (Elliott et al., 2016). Successful regeneration of bone is provided by mechanical stability and a favorable biological microenvironment.

The gold standard treatment for delayed union and non-union involves bone grafts and fixation techniques, which provide a stable environment for the bone to regenerate and bridge the non-union site. In general, autologous bone grafts are often used due to their osteoconductive, osteoinductive, and osteogenic properties, which support bone regeneration (Dimitriou et al., 2011; Costa et al., 2020). Also, in more complex non-union cases, bone stimulators, such as electrical or ultrasound may be considered an adjunct to the treatment (Kolade et al., 2020).

The clinical and surgical problem of bone healing has stimulated researchers and orthopedic surgeons to seek new treatments for successful bone healing. In this context, the number of orthobiologics therapies in the orthopedic field is growing (Dhillon and Patel, 2022). The term orthobiologics refers to a class of regenerative medicine which uses biological (natural) substances to manage musculoskeletal injuries and degeneration; to relieve pain and symptoms; to improve healing after orthopedic surgery; or in cases of ligament or tendon strain, bone or cartilage injuries (Rodriguez-Merchan and Moreno-Garcia, 2021). Then, to address this challenge, biological therapies such as platelet-rich plasma (PRP), bone morphogenetic proteins (BMPs), and autologous mesenchymal stromal cells derived from bone marrow (MSCs) have been introduced with different results (Gálvez-Sirvent et al., 2020; Ho-Shui-Ling et al., 2018).

A recent review from Jamal et al. suggests that PRP may play a clinical role in bone healing (Jamal et al., 2022). Fractures that exhibit compromised biological conditions, leading to inadequate healing, can benefit from therapies that enhance the biological potential at the site of the fracture, referred to as orthobiologics (Emara, 2015). An ideal treatment for bone non-union would provide mechanical stability, biological healing, good functional outcomes, no need for reintervention, and no complications. Currently, there is no such treatment for these types of lesions. Hence, this review assesses whether orthobiologics can boost bone repair to provide a valid tool to improve tissue healing.

The aim of this systematic review is to examine the current literature about the usage of orthobiologics in bone aseptic delayed unions and non-unions in adults' long bones in order to understand the safety and the efficacy of this therapy.

## 2 Materials and methods

**Fig. 1:**
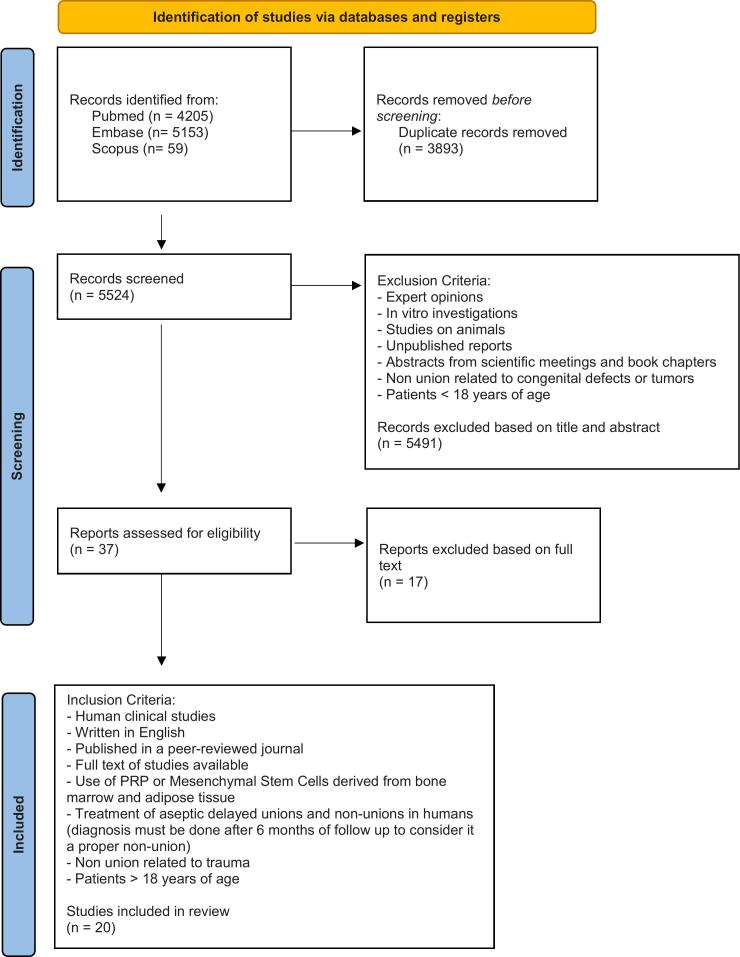
PRISMA 2020 flow diagram for new systematic reviews.

This systematic review is based on the Preferred Reporting Items for Systematic Review and Meta-Analyses (Fig. 1) checklist structure and follows the recommendations of the Enhancing the Quality and Transparency of health Research Network. Moreover, this systematic review was registered in the International Prospective Register of Systematic Review (CRD42023447082).

### 2.1 Focused question

This systematic review was conducted to answer the following question: Is the usage of orthobiologics effective in long bone delayed unions and non-unions in adults?

### 2.2 Search strategy

A literature search was performed among three databases (PubMed, Embase and Scopus) with no date restriction, but limited to publications in the English language. The search was carried out up to May 12, 2023, and was performed with medical subject headings terms/entry terms as follows: “(Platelet-rich plasma OR PRP OR platelet concentrate OR platelet-rich therapy OR platelet gel OR PRF OR adipose-derived stem cells OR stem cells OR adipose stem cells OR bone marrow stem cells OR ASC OR ADSC OR BMSC) AND (non-union OR delayed union OR bone healing OR pseudoarthrosis OR bone defect OR malunion).” In addition, an independent manual search was conducted by using terms adapted for each database, including the grey literature and relevant journals in the field. The manual search was also conducted on the reference lists of relevant review studies. Alerts were established for each database to maintain the search strategy up to date.

### 2.3 Eligibility criteria

The PICO framework (Miller and Forrest, 2001) was used to target our focused question as follows:

(P) population: humans (>18 y.o.) affected by delayed union or non-union secondary to trauma and fracture;
(I) intervention: usage of orthobiologics (PRP, bone grafts, growth factors, ADSCs, BMSCs), for treatment of bone delayed union and non-union.
(C) comparison: fixation revision and/or bone grafts without orthobiologics;
(O) outcome: clinical, biomechanical, and imaging analysis (X-ray and CT scan).

Included in this systematic review were: (1) human clinical studies, (2) articles written in English and (3) published in a peer-reviewed journal with (4) full text available, (5) studies in which PRP or mesenchymal stem cells derived from adipose tissue or bone marrow were used (6) for the treatment of aseptic delayed unions and non-unions in humans adults >18 y.o. (diagnosis must be done after 4 months of follow-up to consider it a delayed union and 6 months of follow-up to consider it a proper non-union).

Studies conducted in animal models or designated as purely in vitro, or human studies on non-union related to patients <18 y.o. or due to congenital defects or tumors, review articles, expert opinions, abstract-only articles, and unavailable full texts were excluded (Fig. 1).

### 2.4 Study selection

For this purpose, all the references retrieved from databases were imported into the Rayyan—Intelligent Systematic Review platform (https://www.rayyan.ai/). Initially, cross-checking eliminated all duplicates, and two reviewers (L.I. and A.P.) independently assessed all titles and abstracts for inclusion using the inclusion criteria described above. In case of a disagreement, a third reviewer (N.R.) was consulted and the final decision was settled by consensus. The kappa coefficient value was calculated to determine interreader agreement. Finally, a full-screen process was performed for the remaining articles that met the inclusion and exclusion criteria.

### 2.5 Data extraction

The following information were recorded: author(s), year of publication, age (years), gender, bone and non-union type, experimental groups, types of orthobiologic used, periods of analysis (months), imaging analysis and main finding. In the case of missing data, one attempt to contact the corresponding author was performed.

## 3 Results

### 3.1 Literature search

The initial literature search identified 9417 studies in total. 3893 duplicate records were removed before screening. A total of 5524 records were screened initially based on the abstract with 37 full-text articles then assessed for eligibility using the inclusion and exclusion criteria. Twenty of these were included in the qualitative synthesis. Seventeen studies, based on full text, were excluded because they did not match the pre-established inclusion criteria (Fig. 1).

### 3.2 Patient demographics & study characteristics

Thirteen out of twenty studies were about non-unions and included 493 patients (63 % M, 37 % F) with an average age of 40.62 years and a mean follow-up of 15.55 months. There were 3 case-control studies (Cen et al., 2022; Duramaz et al., 2018; Wang et al., 2019a), 1 randomized clinical trial (Acosta-Olivo et al., 2017), 3 case series (Sanchez et al., 2009; Mariconda et al., 2008; Tarallo et al., 2012), and 6 prospective studies (Malhotra et al., 2015; Galasso et al., 2008; Calori et al., 2008; Bielecki et al., 2008; Chiang et al., 2007; Singh et al., 2013).

Seven studies were about delayed unions and included 256 patients (70 % M and 30 % F) with an average age of 41.7 years and a mean follow-up of 8.07 months. There were 2 randomized controlled trial (Acosta-Olivo et al., 2017; Samuel et al., 2018), 2 prospective studies (Bielecki et al., 2008; Gołos et al., 2014) and 3 case series (Canton et al., 2023; Ranjan et al., 2023; Say et al., 2014). Participant demographics are reported in Table 1.

**Table 1:** Demographics and follow-up.

Mean	Non-union	Delayed Union
Patients (n)	493	256
Age (years)	40,62 ± 5,06	41,7 ± 8,93
Gender (%M)	311 (63 %)	179 (70 %)
Gender (%F)	182 (37 %)	77 (30 %)
Follow-up	15,55 ± 10,62	8,07 ± 3,60

### 3.3 Management of fracture delayed union

The 7 selected studies that described the role of orthobiologics in the management of delayed union included 256 patients (mean age 41.7 years, 70 % M and mean follow-up of 8.07 months). Localization of long bone non-union is reported in Table 2. In particular, the most common localization was tibia (86) and femur (80). Following other frequent localization were the humerus (44), forearm (34) and fibula (3).

**Table 2:** Non-union localizations.

Localisation	Non-union	Delayed union
Femur	119 (24,5 %)	80 (32 %)
Tibia	181 (37,2 %)	86 (35 %)
Fibula	1 (0,2 %)	3 (1 %)
Humerus	96 (19,8 %)	44 (18 %)
Radius	27 (5,5 %)	.
Ulna	50 (10,3 %)	.
Radius + ulna	9 (1,9 %)	34 (14 %)
Supracondylar	1 (0,2 %)	.
Calvicle	1 (0,2 %)	.
Metacarpal	1 (0,2 %)	.

All the included studies reported radiological and clinical outcomes, including subjective functional scores or clinical examination. Four of the seven studies reported PRP as an orthobiologic type (Acosta-Olivo et al., 2017; Gołos et al., 2014; Ranjan et al., 2023; Say et al., 2014), one Platelet Concentrate (PC) (Samuel et al., 2018), one Platelet-Leukocyte-Rich Gel (PLRG) (Bielecki et al., 2008), one Bone Marrow Aspirate Concentrate (BMAC) (Canton et al., 2023) (Table 4).

The mean follow-up for studies using PRP as an orthobiologic type was 8.8 months, 3 months in the PC group, 6 months in the study about PLRG, and 6.5 months in the BMAC group (Table 4).

In the group of PRP as an orthobiologic type, Ranjan et al. (Ranjan et al., 2023) augmented the fracture site with 3 doses of PRP fluoroscopic guided injection at an interval of 3 weeks, positioning an external stabilization as routine management of fractures, while Golos et al. (Gołos et al., 2014) performed the procedure one time. Say et al. (Say et al., 2014) studied the effect of 3 PRP injection, 1 per week, at mean distance of 6 months from the trauma. Carlos-Acosta et al. (Acosta-Olivo et al., 2017) compared internal fixation with iliac crest graft (ICA) with the same surgery plus PRP intraoperative augmentation.

The study from Samuel et al. (Samuel et al., 2018) has the objective of determining the bone consolidation time among patients with delayed diaphyseal fractures who were managed with PC injection under image intensifier guidance for 2 times in 3 weeks. Bielecki et al. (Bielecki et al., 2008) studied 12 cases of long bone delayed union treated with PLRG percutaneous injection, while Canton et al. (Canton et al., 2023) described the use of BMAC supplementation to ICA in addiction to internal (9 patients) and external (2 patients) fixation.

### 3.4 Management of fracture non-union

All 13 selected studies described the role of orthobiologics in the management of non-union fractures with 493 participants (mean age 40.62 years, 63 % M and mean follow-up of 15.55 months). Localization of long bone non-union is reported in Table 2. In particular, the most common localization was tibia (181) and femur (119). Following other frequent localization were the humerus (96), ulna (50) and radius (27). Only one article (Bielecki et al., 2008), describes non-union localization in one fibula and one clavicle, while Sachez et al. (Sanchez et al., 2009) reported four non-unions in supracondylar bone fractures. Only one metacarpal localization was described (Singh et al., 2013) and nine forearm (radius + ulna) were reported in 3 articles (Wang et al., 2019a; Mariconda et al., 2008; Malhotra et al., 2015).

All the included studies reported radiological and clinical outcomes, and five of the thirteen reported subjective functional scores and/or clinical examination. Nine of the thirteen studies reported PRP as an orthobiologic type (Cen et al., 2022; Duramaz et al., 2018; Acosta-Olivo et al., 2017; Sanchez et al., 2009; Mariconda et al., 2008; Tarallo et al., 2012; Galasso et al., 2008; Calori et al., 2008), one Platelet Rich in Growth Factor (PRGF) (Sanchez et al., 2009), one Platelet-Leukocyte-Rich Gel (PLRG) (Bielecki et al., 2008), one Bone Marrow (Singh et al., 2013), one BMMSC (Wang et al., 2019a) and one Autologous Platelet (Chiang et al., 2007) (Table 3).

**Table 3:** Synopsis of delayed union studies included in the present review.

Publication	Study type (LoE)	Total patients	Age	Pathology	Therapeutic protocol	Protocol specifics	Orthobiologic type	FU (months)	Main findings
Bielecki T. et al., Eur Surg Res. (2008)	Prospective study (IV)	12 (8 M, 4 F)	19–60 (mean 41,4)	Long bone delayed union: 9 tibias and 3 fibulas	PLRG injection	An 18-gauge or biopsy needle was introduced immediately into the gap of delayed union or nonunion under fluoroscopic guidance. In all cases, PLRP and thrombin solution (a total of 15 mL) was injected by dual syringe applicator system (Biomet Inc.) into the disturbed bone-healing area forming a gelatinous mass	Platelet-Leukocyte-Rich Gel (PLRG)	Day 3 as well as 3, 5, 8, 12, 18 and 24 weeks after percutaneous PLRG injection.	In the delayed union group, the average time to union was 9.3 weeks after PLRG injection, and union was achieved in all cases. In the nonunion group, union was observed in 13 out of 20 cases, with an average time to union of 10.3 weeks after PLRG injection. Interestingly, in patients where union was not achieved, the average time from the fracture and/or the last operation was over 11 months. Fortunately, no complications were observed.
Golos et al., Ortopedia Traumatologia (2014)	Prospective study (IV)	132 (79M, 53F)	18–85 (mean 41)	Long bone delayed union: 21 humerus, 32 forearms, 23 femurs, 47 tibias	PRP fluoroscopic guided injection	The patients with diagnosed delayed bone union had platelet rich plasma administered into the fracture cleft. The procedure was performed under radio- graphic guidance with local by the closed percutaneous method	PRP	Radiographs were obtained every 6 weeks until bone union was observed.	Bone union was observed in 108 patients (81.8 %) following PRP administration. The treatment demonstrated its highest efficacy in patients with delayed union of the proximal tibia who underwent surgical intervention with open reduction and plate fixation (100 % success rate), typically achieving union after an average of 3.5 months post-PRP administration. Conversely, the lowest efficacy was noted in patients with delayed union of the proximal humerus who underwent surgical intervention with open reduction and plate fixation (63.64 % success rate), typically achieving union after an average of 3.2 months post-PRP administration.
Say et al., Acta Chirurgiae Orthopaedica (2014)	Retrospective case series (IV)	20 (17M, 3F)	Age 33.5 range 18–77	Long bone non union: 12 Long bone delayed union: 8 (16 femurs and 4 tibias in total)	PRP fluoroscopic guided injection	The prepared PRP was injected into the fracture line under fluoroscopy guidance for totally three times once a week. The application of PRP was made at median 6 (range 6–8) months after fracture surgery.	PRP	Median period of 11 (range 8–12) months	During the follow-up period, eleven patients experienced non-union of the fracture and required revision surgery. Radiological and clinical evidence of sufficient union was not observed in three patients. Among the delayed union group, six out of eight patients achieved fracture union. Notably, no patient in the non-union group attained fracture union.
Carlos Acosta-Olivo et al., Arch Orthop Trauma Surg (2017)	RCT (I)	16 (13M, 3F)	21–60 (mean 38.1)	Humerus delayed union	ICA (9) vs ICA + PRP (7)	LCP fixation with an ICA was performed in the control group; this treatment was supplemented with the intraoperative administration of 12 mL of autologous PRP in the study group. All the patients were subjected to the same initial surgical procedure.	PRP	2, 4, 6, 12, 24, and 36 weeks of evolution.	Patients treated with PRP showed an earlier onset of bone consolidation, with signs evident at 2 weeks compared to 6 weeks in the control group. Additionally, these patients achieved bone consolidation at an average of 19.9 weeks, whereas the control group required 25.4 weeks on average. Importantly, the experimental group achieved union in 100 % of cases, with only one patient failing to achieve union. Clinical scores were similar between the two groups.
Samuel G. et al., European Journal of Orthopaedic Surgery & Traumatology (2018)	RCT (I)	40 (39M, 1 F)	20–60 (mean 37)	Long bone delayed union (Femurs 29, Tibias 8, Forearms 2, Humerus 1)	PC 23 (percutaneous injection under fluoroscopic guidance) vs Observation 17	Before injection the prepared PC is activated by adding 10 % calcium gluconate in the ratio 3:10, following which the PC is loaded into a 10-mL syringe and injected percutaneously at the delayed union site under image intensifier guidance. The study group patients are given a second and final PC injection at the delayed union site at 3 weeks from the first injection by repeating the same procedure.	PC (Platelet Concentrate)	Every 6 weeks until fracture union	The percentage of union was 78 % (18 out of 23) in the PC group and 59 % (10 out of 17) in the control group (*p* = 0.296). The mean time to fracture union treated with PC (15.33 ± 9.91 weeks) did not differ significantly from the control group (13.10 ± 7.21 weeks; *p* = 0.540). In the PC group, union was observed in 60 % of cases after 12 weeks following PC injection.
Canton et al., Acta Biomed (2023)	Case series (V)	11 (4M, 7F)	46–84 (mean 61)	Long bone delayed union and 8 non union (femurs 36 %, tibias 45 %, other long bones where humerus and clavicle).	Surgery + BMAC	2 patients were treated with circular external fixator and percutaneous injection of BMAC alone. All other patients received the combination of internal fixation (plate/nail) and cancellous allograft with BMAC supplementation	BMAC	6,5 months (until union occurred)	All 11 patients (100 %) achieved union after treatment, with a mean time to radiographic union of 6.5 months (range 4–12). Notably, none of the patients experienced complications during the evaluation period. Specifically, there were no instances of donor site morbidity, hematoma, or wound complications at the iliac crest BMAC harvesting site.
Ranjan R. et al., Journal of Orthopedics (2023)	Case series (V)	25 (19M, 6F)	29–63 (mean 40)	Long bone delays union (7 femurs, 4 humerus, 14 tibias)	PRP (fluoroscopic guided injection)	Fractures were augmented with 3 doses of autologous PRP injection with each dose being administered at an interval of 3 weeks. Under c-arm guidance, the delayed union site was localized and an autologous PRP solution is infiltrated. After administering an autologous PRP injection at the fracture site, an external stabilization was given as per the routine management of fractures.	PRP	12 months	Out of 25 cases, 21 (84.00 %) showed good union of the fracture with adequate callus formation within 10–12 weeks after receiving 3 doses of autologous PRP injections. The mean pre-procedural VAS and Warden's score at the final follow-up revealed statistically significant improvements (*p* < 0.05). Throughout the study period, no other complications were attributed to autologous PRP application among the study participants, except for 3 cases, which included 2 instances of non-union and 1 case of implant failure.

The mean follow-up for studies using PRP as an orthobiologic type was 14.85 months. Specifically, 30 months in the group that used PRGF, 6 months in the group that used PLRG, 32.4 months in the group that used autologous platelet gel, 6 and 9 months respectively, in bone marrow and BMMSCs groups (Table 3).

In the group of PRP as an orthobiologic type, Cen et al. (Cen et al., 2022) compared PRP with PRP + ESW (Extracorporeal Shock Waves), while Duramaz et al. (Duramaz et al., 2018) compared in patients treated for the first time with closed-reamed intramedullary nailing, who had developed a long bone non-union (15 femurs and 14 tibias), the use of PRP with the exchange intramedullary nail. Mariconda et al. (Mariconda et al., 2008) compared the use of PRP 2 days before external fixation with the only use of external fixation, while Malhotra et al. (Malhotra et al., 2015) compared seventy-one patients who had previously undergone open reduction and internal fixation with 23 patients who were being treated by closed reduction and plaster application. All patients received 15–20 mL of autologous platelet-rich plasma under an image intensifier. The study from Acosta-Olivo et al. (Acosta-Olivo et al., 2017) has the objective of determining the bone consolidation time among patients with non-union diaphyseal humeral fractures who were managed with locking compression plate (LCP) fixation combined with an iliac crest autograft (ICA) using PRP as a co-adjuvant. Tarallo et al. (Tarallo et al., 2012) studied 10 cases of ulna non-union, treated with osteosynthesis using a dynamic compression plate and biological enhancement of the consolidation using bone graft and autologous platelet injection. Galasso et al. (Galasso et al., 2008) describe 22 cases of long bone non-union, in which Patients were treated with removal of pre-existing hardware, decortication of non-union fragments, and fixation of pseudoarthrosis with expandable intramedullary nailing; PRP was placed in the pseudoarthrosis rim. In the last case of PRP used as an orthobiologic type, Calori et al. (Calori et al., 2008) compared the use of PRP in 60 patients with long bone non-union with rhBMP-7 in other 60 patients.

Sanchez et al. (Sanchez et al., 2009) described the use of PRGF as an orthobiologic; 13 patients were treated surgically (nail in diaphyseal non-union and plate in supracondylar non-union) + PRGF/graft application, while 3 patients were treated with percutaneous injection of PRGF without exposing the fracture site (3 injections in 6 weeks).

Only one study takes into consideration the use of Platelet-Leucocyte-Rich Gel as an orthobiologic (Bielecki et al., 2008), with direct injection of the PLRG under fluoroscopic guidance in the non-union site and one described the treatment of 12 cases of long bone non-union with Bone graft enriched with APG (Chiang et al., 2007).

The last two studies describe the use of Bone Marrow injection. The first one used BMSC aspired from the anterior iliac crest in 12 cases of long bone non-union where the orthobiologic was injected into the delayed and non-union sites under fluoroscopy (Chiang et al., 2007). The second one (Wang et al., 2019a) compared the Iliac crest autograft with a novel system called the bone marrow stem cell Screen-Enrich-Combine Circulating System (SECCS).

### 3.5 Main findings

The main findings are reported in Table 3, Table 4. In general, the use of orthobiologics led to better results when compared to other surgical procedures that didn't include the injection of biological factors (Duramaz et al., 2018; Mariconda et al., 2008).

**Table 4:** Synopsis of non-union studies included in the present review.

Publication	Study type (LoE)	Total patients	Age	Pathology	Therapeutic protocol	Protocol specifics	Orthobiologic type	FU (months)	Main findings
Cen C. et al., Orthop Traumatol Surg Res. (2022)	Case-control (III)	55 (31 M, 29 F)	18–60	Long bone non union: 18 tibias, 15 femurs, 9 humerus, 6 radii, 12 ulnae	PRP (27) vs PRP + ESW (28)	PRP was directly injected into the nonunion area once a week for 3 weeks. PRP was injected for 2 days before shock wave therapy was determined to be used in the PRP + ESW group. The shock wave therapy was performed once a week, 3 times as a course of treatment.	PRP (platelet concentration of 420 %) (mean: 780,000 platelets/mL)	16–18	The fracture union rate was 92.59 % in the PRP + ESW group compared to 71.43 % in the PRP group. The clinical healing time was significantly longer in the PRP group than in the PRP + ESW group (*p* < 0.05). Additionally, the Johner-Wruhs functional classification was lower in the PRP group than in the PRP + ESW group.
Altuğ Duramaz et al., Eur J Orthop Surg Traumatol (2017)	Case-control (III)	29 (16M, 13F)	35.14 ± 11.83 years in PRP group and 41.8 ± 8.18 years in the control group	Long bone non union: 15 femurs, 14 tibias	PRP (14) vs Exchange intramedullary nail (15)	In all patients, the first treatment modality was closed reamed intramedullary nailing. In control group the nail was changed with a larger one. Before exchange nailing, the intramedullary canal was reamed. In PRP group percutaneous PRP was performed with PRP applicator under fluoroscopy.	PRP (2,000,000/μl activated thrombocyte in quality control of the preparation)	34.97 ± 8.78 months in PRP group and 33.73 ± 10.53 months in the control group	The average healing time was shorter in the PRP group at 16.71 ± 2.4 weeks compared to 19.07 ± 3.67 weeks in the EIN group (p = 0.053). At the conclusion of the follow-up, union was achieved in 92.8 % of cases in the PRP group, while this ratio was 80 % in the control group. The mean VAS values during both the preoperative and postoperative periods did not show statistically significant differences in either group (p > 0.05).
Mikel Sanchez et al., J Orthop Trauma (2009)	Retrospective case series (IV)	15	Mean age 46.3	Long bone non union: 12 diaphyseal (4 humerus, 4 femurs, and 4 tibias) and 4 supracondylar	PRGF (3) vs surgery + PRGF (13)	13 patients were treated surgically (nail in diaphysial non union and plate in supracondylar non union) + PRGF/graft application. 3 patients were treated with percutaneous injection of PRGF without exposing the fracture site (3 injections in 6 weeks).	PRGF (it can be injected without surgery or it can be mix with the morselized bone allograft)	12–48 months	All nonunions that were treated operatively achieved healing after a single procedure, although two patients required additional PRGF injections. In cases of stable nonunions, two out of three achieved healing only after repeated percutaneous PRGF injections. The average time from surgery and/or PRGF application to union was 4.9 months (ranging from 2 to 8 months). No complications associated with the described procedure were observed.
Massimo Mariconda et al., J Orthop Trauma (2008)	Prospective case series with historical controls. (IV)	40 (22M, 18F)	30.2 6 14.1	Long bone non union: 24 tibia, 12 humerus, 4 forearms	External fixation + PRP (20) vs external fixation (20)	A MMF device (Amplimedical, Assago, Milano, Italia) was used in all patients. One- centimeter fibula resection was also carried out to obtain interfragmentary compression on 12 patients being treated for tibial nonunion. During surgery, PG was percutaneously injected in the interfragmentary space under fluoroscopic guidance.	PRP withdrawn 2 days before surgery and frozen (1,075,020 platelets/mL)	9 months (in order to judge non union)	The healing rate was 90 % (18 out of 20 cases) in platelet gel cases and 85 % (17 out of 20 cases) in the control group (P = 0.633). The mean time until radiographic consolidation in the PRP group (147 ± 63 days) did not differ significantly from that in the control group (153 ± 61 days; P = 0.784). When analyzing the mean healing time for separate segments, no differences were noted between the study and control groups.
R. Malhotra, Musculoskelet Surg (2015)	Prospective study (IV)	94 (66M, 28F)	Unknown	Long bone non union: 35 tibia, 30 femur, 11 humerus, 4 radius, 12 ulna, 2 with both radius and ulna	Injection of PRP	Seventy-one patients had previously undergone open reduction and internal fixation, while 23 patients were being treated by closed reduction and plaster application. All patient received 15–20 mL of autologous platelet-rich plasma under image intensifier.	PRP (2,000,000 platelets/ll)	4 months	At the end of 4 months, 82 patients had their fractures united. Bridging trabeculae were observed on X-rays in 34 patients at the end of 2 months, while 41 patients showed bridging trabeculae by the end of the third month. Twelve patients did not exhibit any signs of union at 4 months and were classified as treatment failures. Fortunately, no complications were reported. Among the patients whose fractures united, platelet injections had been administered within 2–4 months of the diagnosis of nonunion.
Carlos Acosta-Olivo et al., Arch Orthop Trauma Surg (2017)	Controlled randomized trial (I)	16 (13M, 3F)	21–60	Humeral shaft non union	Iliac crest allograft (9) vs ICA + PRP (7)	LCP fixation with an ICA was performed in the control group; this treatment was supplemented with the intraoperative administration of 12 mL of autologous PRP in the study group. All the patients were subjected to the same initial surgical procedure.	PRP	8 months	The mean bone consolidation time for the ICA group was 25.44 ± 2.06 weeks, while for the ICA + PRP group, it was significantly lower at 19.9 ± 2.25 weeks (P < 0.05). All patients in the ICA + PRP group achieved bone consolidation, whereas in the ICA group, only one patient did not achieve bone consolidation by the end of the follow-up period. At week 2, the quick-DASH score was 76.41 ± 19.60 for the ICA group and 81.50 ± 9.04 for the ICA + PRP group. A significant decrease in the quick-DASH score was observed from week 4 (p < 0.05) up to week 36 (p < 0.001) in both groups.
Tarallo L. et al., Eur J Orthop Surg Traumatol (2012)	Case series (V)	10 (7M, 3F)	25–50	Ulna non union (7 after ORIF, 2 after nailing, 1 after cast)	PRP injection	Two days before surgery, 450 cm^3^ of venous blood was obtained from each patient in order to prepare PRP. Surgery: direct posterior incisions, excision of all fibrous issue and sclerotic bone from the fracture site, Nicoll's technique modified by Davey and Simonis, the defect is completely filled with bone, and over it is placed a 3.5 mm LC-DCP. Injection of PRP in the non-union site.	PRP	21 months (7–40)	Bony union was achieved in 9 out of 10 cases, on average within 4 months. During follow-up, the mean VAS score for pain in the upper limb was 1 at rest (ranging from 0 to 4) and 2 during activities (ranging from 0 to 7). The physical function and symptoms of the upper limb, assessed with the DASH questionnaire, scored 17 points. Importantly, none of the 10 patients experienced issues related to weakness or instability of the elbow after treatment.
O. Galasso et al., J Orthopaed Traumatol (2008)	Prospective study (IV)	22 (13M, 9F)	Mean 39 (20–56)	Long bone non union: tibia 11, femur 8, and humerus 3	Nailing + PRP	Patients were treated with removal of pre-existing hardware, decortication of non-union fragments, and fixation of pseudoarthrosis with expandable intramedullary nailing. At surgery, PRP was placed in the pseudoarthrosis rim.	PRP	13 months	Ninety-one percent (20 out of 22 patients) achieved bony union, with an average time to union of 21.5 weeks. There were no reported instances of infection, neurovascular complications, rotational malalignment, or limb shortening. However, two cases, one involving the femur and the other the tibia, failed to consolidate. Regarding functional outcomes, 17 out of 19 patients who initially had pseudoarthrosis of the lower limb were able to walk without any support at the final follow-up. Additionally, 12 out of 22 patients returned to sports activities.
G.M. Calori et al., Injury, Int. J. Care Injured (2008)	Prospective randomized clinical study (I)	120 (67M, 53F)	Mean 42 (19–65)	Long bone non union. rhBMP-7 group: 15 tibias, 10 femurs, 15 humerus, 12 ulnas, and 8 radii. PRP group: 19 tibias, 8 femurs, 16 humerus, 8 ulnas, and 9 radii.	rhBMP-7 (60) vs PRP (60)	The only rhBMP available in Italy is rhBMP-7 mixed with a bio-reabsorbable carrier (3.5 mg Eptotermin alpha, +1 g collagen, Osigraft) Patient blood whit drawn was used to obtain 20 mL of PRP. Revision of the previous fixation plus homologous bone preserved from our bone bank was used in order to fill larger segment gaps or Xenografts and synthetic bone substitutes (hydroxyapatite) were used mainly as fillers for small bone defects.	rhBMP and PRP	12.43 months (range 9–25 months).	Both clinical and radiological union occurred in 52 (86.7 %) cases of the rhBMP-7 group compared to 41 (68.3 %) cases of the PRP group. The rhBMP-7 group exhibited a lower median clinical and radiographic healing time (3.5 months vs. 4 months and 8 months vs. 9 months, respectively). However, complications arose in both groups, with four non-unions in the rhBMP-7 group and five non-unions in the PRP group complicated by infection. Despite adjuvant treatment, these cases failed to progress to union. For the remaining three cases of the rhBMP-7 group and the thirteen cases of the PRP group, a re-intervention procedure was deemed necessary.
T. Bielecki et al., Eur Surg Res (2008)	Prospective study (IV)	20 (16M, 4F)	Mean age 39,45	Long bone non union: 3 humerus, 2 femurs, 11 tibias, 2 radius,1 fibula, 1 clavicula	PLRG injection	The surgical procedure was performed in the operating room under general anesthesia. An 18-gauge or biopsy needle was introduced immediately into the gap of delayed union or nonunion under fluoroscopic guidance.	Platelet-Leukocyte-Rich Gel (PLRG)	6 months	Union was observed in 13 out of 20 cases, with an average time to union of 10.3 weeks after PLRG injection. In patients where union was not achieved, the average time from the fracture and/or the last operation was 11 months. In the non-union group, factors such as the number of fracture site operations, type of nonunion, and fracture localization did not significantly influence treatment results. However, the crucial factor appeared to be the average time from the initial surgery to PLRG injection for nonunion; 11 months seemed to be critical for favorable outcomes.
Chao-Ching Chiang et al., J Trauma. (2007)	Prospective study (IV)	12 (9M, 3F)	Mean age 50.5 (22–86)	Long bone non union: 8 tibias, 4 femurs	Bone graft enriched with APG	Patients were treated with the necessary procedures as indicated by their individual problems, including removal of previous implants, debridement, soft tissue reconstruction, fixation with internal or external fixators, and bone graft enriched with APG.	Autologous Platelet Gel (APG)	32,4 months	Out of the 12 patients, 11 healed at an average of 19.7 weeks after the first attempt, and 1 healed after the second attempt at 21 weeks. Throughout the healing process, bone mineral density steadily increased from the early healing stage to the remodeling phase. Additionally, functional status showed significant improvement, with patients experiencing enhanced function at an average follow-up of 32.4 months.
Ashok K Singh et al., Journal of Orthopaedic Surgery (2013)	Prospective study (IV)	10 (5M, 5F)	Mean 45	Long bone non union: 6 ulnas, 3 femurs, 2 humerus, 1 metacarpal.	Bone marrow injection	The mean time since injury was 9 (range, 5–53) months. Bone marrow was aspirated from the anterior iliac crest and injected to the delayed and non-union sites under fluoroscopy. Three injection were performed. The interval between the injections was 6 to 8 weeks. The amount of bone marrow injected was 30 to 40 mL for long bones and 20 mL for metacarpals.	Bone marrow	6 months	Nine out of ten non-unions of bones healed after bone marrow injections. The mean time for callus formation was 5.8 weeks (range, 3–10 weeks), for clinical union was 7 weeks (range, 4–12 weeks), and for radiological union was 16 weeks (range, 10–24 weeks).
Xin Wang et al., Cell Transplantation (2018)	Retrospective case-control (III)	50 (38M, 12F)	36.0+-14.3 and 43.1+13.9	Long bone non union: 23 femurs, 19 tibias, 5 humerus, 3 forearms.	30 SECCS therapy vs 20 AGT	The AGT group underwent iliac crest autograft. SECCS group underwent implantation of a novel system called the bone marrow stem cell Screen-Enrich-Combine Circulating System (SECCS) by seeding mesenchymal stem cells (MSCs) into b-tricalcium phosphate (b-TCP) during surgery to thereafter rapidly process bioactive bone implantation. By SECCS therapy, the MSC-enriched b-TCP particles were implanted into the non-union gap. During the enrichment procedure, a significant proportion of MSCs were screened and enriched from bone marrow into porous b-TCP particles	BMMSCs (11,444.0 + 6018 cells were transplanted per patient)	9 months	After 9 months of follow-up, 27 patients (90 %) in the SECCS group achieved clinical union, compared to 18 patients (90 %) in the AGT group (p = 0.064 for clinical union time). Additionally, the postoperative radiographic union score at 9 months post-operation was similar between the two groups.

With regards to the delayed union studies, a summary is reported in Table 3.

Bielecki et al. (Bielecki et al., 2008) demonstrated that in the delayed union group, PLRG injection led to successful union in all cases with an average time of 9.3 weeks, while in the nonunion group, union was observed in 65 % of cases at an average time of 10.3 weeks. Notably, patients who did not achieve union had a prolonged average time from the fracture or last operation exceeding 11 months, highlighting the impact of timing on treatment efficacy. Samuel et al. (Samuel et al., 2018) study comparing PC injection group and control group showed a higher percentage of union in the PC group (78 % vs. 59 %) without significant differences in mean time to fracture union. In the PC group, 60 % achieved union within 12 weeks of injection, indicating a potential for expedited recovery. Canton et (Canton et al., 2023) al reported a 100 % union rate in patients treated with allograft augmented with BMAC, with a mean time to radiographic union of 6.5 months and no complications.

Golos et al. (Gołos et al., 2014) investigation into PRP injection revealed an overall bone union rate of 81.8 %, with the highest efficacy seen in surgically treated proximal tibia delayed union cases (100 %) at 3.5 months post-PRP, contrasting with the lowest efficacy in proximal humerus cases (63.64 %) at 3.2 months. Ranjan et al. (Ranjan et al., 2023) demonstrated an 84 % union rate with autologous PRP fluoroscopig guided injections, showing statistically significant improvements in clinical scores. Complications were limited to three cases (2 non-unions and 1 implant failure) among 25 participants.

Carlos Acosta et al. (Acosta-Olivo et al., 2017) exploration of PRP supplementation of ICA in humerus fractures open reduction and internal fixation, showed an accelerated onset of bone consolidation at 2 weeks, leading to a union in 100 % of cases at an average of 19.9 weeks, compared to 25.4 weeks in the control group. Say et al. (Say et al., 2014) reported on cases of non union, necessitating revision surgery in 44 % of patients, while 75 % of patients in the delayed union group achieved fracture union.

Main findings of non-union fractures studies are shown in Table 4.

Duramaz et al. (Duramaz et al., 2018) demonstrated that the mean healing time was shorter in the PRP group (16.71 ± 2.4 weeks) when compared with the exchange intramedullary nail group (19.07 ± 3.67 weeks) (*p* = 0.053). At the end of the follow-up, the union was achieved in 92.8 % of the cases in the PRP group. This ratio was 80 % in the control group. The mean VAS values in preoperative and postoperative periods were not statistically significant in both groups (*p* > 0.05).

Mariconda et al. (Mariconda et al., 2008) demonstrated that the healing rate was 90 % (18/20) in patients who underwent external fixation plus platelet gel injection and 85 % (17/20) in who underwent external fixation alone (*p* = 0.633). The mean time until radiographic consolidation in PRP group (63 days) was not different to the result in the control group (61 days; *p* = 0.784). Analyzing the mean healing time for separate segments, no differences were noted between the experimental and control group.

PRP resulted in empowering the biological environment in which the implant is placed. Galasso et al. (Galasso et al., 2008) followed 22 patients in a prospective study treating their long bone non-union with the removal of pre-existing hardware, decortication of fragments, and fixation of pseudoarthrosis with expandable intramedullary nailing. At surgery, PRP was placed in the pseudoarthrosis rim. The 91 % (20/ 22 patients) of patients obtained bony union. The average time to union was 21.5 weeks. Only two non-unions, 1 femur and 1 tibia, failed to consolidate. As for the functional outcomes, 17 out of 19 patients originally suffering from pseudoarthrosis of the lower limb were able to walk without any support at the final follow-up. 12 out of 22 returned to sports practice. Tarallo et al. (Tarallo et al., 2012) observed that using PRP in 10 ulnas non-union surgical cases, the bony union was achieved in 9/10 cases on an average time of 4 months. At follow-up, the mean VAS score for pain in the upper limb was 1 (range, 0–4) at rest and 2 (range, 0–7) during activities. The physical function and symptoms of the upper limb, evaluated with the DASH (Disability of the Arm, Shoulder, and Hand) questionnaire, scored 17 points. None of the 10 patients experienced problems with weakness or instability of the elbow after treatment.

Acosta-Olivo et al. (Acosta-Olivo et al., 2017) compared the use of the iliac crest autograft and the use of the iliac crest autograft plus PRP. Their study demonstrated that the mean bone consolidation time in the ICA + PRP group was significantly lower (19.9 ± 2.25 weeks vs 25.44 ± 2.06 weeks, *P* > 0.05). Moreover, bone consolidation was achieved in all patients from the ICA + PRP group, meanwhile, in the ICA group, one patient did not achieve bone consolidation at the end of the follow-up. Regarding the functional results, the quick-DASH score was 76.41 ± 19.60 for the ICA group and 81.50 ± 9.04 for the ICA + PRP group at week 2. A significant decrease in the quick-DASH score was observed from week 4 (*p* < 0.05) up to week 36 (*p* < 0.001) in both groups. In addition, Chao-Ching Chiang et al. (Chiang et al., 2007) with their prospective study demonstrated the efficacy of the enrichment of autologous bone graft with autologous platelet gel when filling a bone defect in a surgical procedure. Of the 12 patients with long bone non-union, 11 healed at an average of 19.7 weeks after the first attempt and 1 healed after the second procedure at 21 weeks. The bone mineral density continued to increase steadily from early healing to the remodeling phase. Functional status was greatly improved at an average follow-up of 32.4 months.

When the implant is mechanically working, orthobiologics are tested by injecting it in the non-union site. Sanchez et al. (Sanchez et al., 2009) observed that out of 15 non-unions, 13 were treated operatively and healed after a single procedure, even though additional PRGF had to be injected in 2 patients. 3 stable non-unions were treated with an injection of PRGF and achieved healing after repeated percutaneous PRGF injections. The mean time from surgery and/or PRGF application to union was 4.9 months (2–8 months). Malhotra et al. (Malhotra et al., 2015) treated 94 patients with PRP injection. 82 of them had their fracture united at the end of 4 months. 34 patients showed bridging trabeculae on X-rays at the end of 2 months, while 41 patients showed bridging trabeculae at the end of the third month. Only 12 patients did not show any attempt of the union at 4 months and were labelled as failure of treatment. Bielecky et al. (Bielecki et al., 2008) studied 20 long bone non-unions after fluoroscopic guided PLRG injection and found that union was observed in 13 of 20 cases and the average time to union was 10.3 weeks after the procedure. Interestingly, in patients in whom the union was not achieved, the average time from the fracture and/or from the last operation was 11 months. The time from the initial surgery to the PLRG injection of 11 months seems to be critical for good outcomes. Ashok K Singh et al. (Singh et al., 2013) found that 9 out of the 10 non-union of long bones healed after bone marrow injections. The mean time for callus formation was 5.8 (range, 3–10) weeks, for clinical union was 7 (range, 4–12) weeks, and for radiological union was 16 (range, 10–24) weeks.

Cen et al. (Cen et al., 2022) tried to understand the role of physical therapy by comparing the use of PRP with PRP plus Extracorporeal Shock Wave (ESW) therapy. In the PRP + ESW group, the fracture union rate was 92.59 % and the healing time was 16.3 ± 5.2 weeks. In the PRP group, the fracture union rate was 71.43 % and the healing time was 21.5 ± 3.7 weeks. The clinical healing time of PRP was significantly longer than in the PRP + ESW (*p* < 0,05) and Johner-Wruhs functional classification in PRP group was lower than in the PRP + ESW group.

In all the studies cited above there were no complications related to orthobiologics use. Only two authors (Calori et al., 2008; Wang et al., 2019b) used less conventional orthobiologics in their studies. Calori et al. (9) compared PRP with rhBMP-7 on 120 long bone non-unions. Both clinical and radiological union occurred in 52 (86.7 %) cases of the rhBMP-7 group compared to 41 (68.3 %) cases of the PRP group, with a lower median clinical and radiographic healing time observed in the rhBMP-7 group (3.5 months vs. 4 months and 8 months vs. 9 months, respectively). Four non-unions in the rhBMP-7 group and five non-unions in the PRP group were complicated by infection and despite adjuvant treatment, they failed to progress to union. For the remaining three cases of the rhBMP-7 group and the thirteen cases of the PRP group, a re-intervention procedure was deemed necessary.

Xin Wang et al. (Wang et al., 2019b) compared Iliac Crest Autograft with a novel system called the bone marrow stem cell Screen-Enrich-Combine Circulating System (SECCS) Therapy. After 9 months of follow-up, 27/30 patients (90 %) in the SECCS group acquired clinical union, compared with 18/20 patients (90 %) in the ICA group (clinical union time, *p* = 0.064) and also post-operative radiographic union score at 9 months post-operation was similar between the two groups.

## 4 Discussion

The treatment of aseptic non-unions or delayed unions is a challenging issue in the orthopedic field. Giannoudis et al. in 2007 described the so-called diamond-shaped concept which refers to the contemporary presence of osteoconductive mediators, osteogenic cells, an osteoconductive matrix (scaffolds), an optimum mechanical environment, an adequate vascularity and, the necessity to address any existing comorbidity of the patient to provide the better substrate for bone healing (Giannoudis et al., 2007).A.s the comprehension of bone healing mechanisms at a molecular scale continues to advance and refine, the favorable modification of the local fracture microenvironment through orthobiologics is progressively emerging as a focal point of interest and therapeutic target in orthopedic surgery.

The most widely studied orthobiologic for bone non-union is Platelet Rich Plasma. PRP is prepared by isolating and concentrating a patient's platelets, which contain growth factors essential for tissue repair. When applied directly to the non-union site, PRP can stimulate the recruitment of mesenchymal stem cells and promote angiogenesis, leading to improved bone regeneration (Foster et al., 2009). Several clinical studies have reported positive outcomes with PRP in the treatment of bone non-union (Patel et al., 2013).

Another promising orthobiologic is Bone Morphogenetic Protein, a naturally occurring protein that plays a crucial role in bone formation. BMP can be delivered in various forms, including recombinant proteins and gene therapy. Studies have shown that BMP can induce osteogenesis, making it a valuable tool in promoting bone healing, particularly in cases of non-union (Urist, 1965). However, its use may be associated with complications such as augmentation of malignancies when used in high doses and should be carefully considered case-by-case (Carragee, 1997).Additionally, Mesenchymal Stem Cell therapy has gained attention as a regenerative approach for bone non-union. MSCs can differentiate into bone-forming cells and modulate the immune response, potentially accelerating bone healing (Caplan, 1991). Clinical trials exploring the effectiveness of MSC-based treatments for bone non-union, or cartilage regeneration are ongoing and showing promising results (Jones et al., 2019; Ulivi et al., 2023).

Despite the vast amount of preclinical data endorsing the utilization of orthobiologics, their success has yet to be mirrored in clinical trials up to this point. For example, animal models, such as rats and rabbits, have been used to simulate non-union fractures. PRP treatments have consistently shown positive outcomes in improved bone callus formation and enhanced fracture stability (Li et al., 2020; Rossi et al., 2023a). These studies often use histological, radiological, and biomechanical assessments to evaluate the effects of PRP on bone regeneration. Additionally, pre-clinical studies have focused on the underlying cellular and molecular mechanisms through which PRP affects non-union fractures. Research has shown that PRP contains a rich milieu of growth factors, such as platelet-derived growth factor (PDGF), transforming growth factor-beta (TGF-β), and vascular endothelial growth factor (VEGF), which play pivotal roles in promoting osteogenesis, angiogenesis, and tissue repair (Foster et al., 2009; Marx, 2001).

While most of the pre-clinical studies have reported favorable outcomes, it is essential to acknowledge the need for further investigations to address remaining questions and optimize PRP therapies. Studies about blood withdraw time from the surgery, PRP preparation and volume to inject into the fracture site should be undertaken. Our review suggests that orthobiologics may have a clinical role in managing bone healing, however, these results are not statistically significant.

The qualitative synthesis comprised thirteen studies on orthobiologics and bone non-unions, encompassing 493 patients with an average age of 40.62 years and a mean follow-up duration of 15.55 months. Various study designs, such as Case-Control, RCTs, Case Series, and Prospective studies, were incorporated into the analysis. Long bone non-union predominantly occurred in the tibia and femur, with the humerus, ulna, and radius being less frequent locations. There were also cases of non-union in other bones like the fibula, clavicle, supracondylar bone, metacarpal bones, and forearm (radius and ulna). On the other hand, only seven studies described the use of orthobiologics in delayed union. Despite the favorable clinical outcomes for the delayed unions treated with orthobiologics, the short follow-up (mean 8.07 months) represents a downside of the studies analyzed. Additional analyses with longer follow-ups are required to confirm the data.

In this systematic review, it was expected to find several Level-1 trials using orthobiologics, however, only 3 randomized clinical trials matched the inclusion criteria. There was heterogeneity in the outcome metrics used in the studies. All the studies used the time to union as an outcome metric with multiple other functional scores employed to assess clinical outcomes. The studies used various types of orthobiologics, and PRP was the most used orthobiologic (8 out of 13 studies). Other orthobiologics included PRGF, PLGF, bone marrow, BMMSCs, and autologous platelet. Each type of orthobiologic had varying mean follow-up times.

Despite our understanding of fracture union principles, addressing non-union and delayed union with biological agents poses a significant challenge. Obstacles to obtain robust evidence in this area include considerable diversity in patient biological characteristics, complexities in injury presentation (such as polytrauma versus isolated injury and fracture patterns), variability in initial fracture management, subjective classification of delayed union and non-union, differences in stem cell application techniques, and the presence or absence of adjuncts. Additionally, there is a dearth of published completed studies.

The heterogeneity of the orthobiologics used in the studies included in this systematic review suggests that a consensus has yet to be defined: what is the best orthobiologic to be used and in what kind of patient and/or fracture is still not known. It must be noted that there is significant uncertainty in PRP composition and a lack of standardization in PRP preparation because there is no literature to support any PRP injection protocol, with uncertainty surrounding optimal dosage and timing intervals (Hurley et al., 2019). Overall, the use of orthobiologics appeared to lead to better results compared to surgical procedures that did not involve the injection of biological factors both for bone non-unions and delayed unions. PRP empowers the biological environment in which the implant is placed. Galasso et al. (Galasso et al., 2008) found that 91 % of patients obtained bony union, and the maximum time to union was 21.5 weeks. Functional scores improved in 89 % of patients. As we know from the literature (Kolade et al., 2020; Rossi et al., 2023b), bone autograft is the gold standard as a biological bone substitute for filling bone defects because of its osteoinductive, osteoconductive, and osteogenic properties. In these cases, orthobiologics are used in order to boost the biological environment. Acosta-Olivo et al. (Acosta-Olivo et al., 2017) compared the use of the Iliac Crest Autograft and the Iliac Crest Autograft plus PRP. Their study demonstrated that mean bone consolidation time in the ICA + PRP group was significantly lower with better functional results. In addition, Chao-Ching Chiang et al. (Chiang et al., 2007), with their prospective study demonstrated the efficacy of the enrichment of autologous bone graft with autologous platelet gel when filling a bone defect in a surgical procedure.

When bone non-union is due to the poor biological environment and the implant is mechanically working, orthobiologics seem to work also when percutaneously injected in the non-union site regardless of the orthobiologic type (Sanchez et al., 2009; Malhotra et al., 2015; Bielecki et al., 2008; Singh et al., 2013). Interestingly, Malhotra et al. (Malhotra et al., 2015) in patients in whom the union was not achieved, the average time from the fracture and/or from the last operation was 11 months. The time from the initial surgery to the PLRG injection of 11 months is critical for good outcomes. Only two authors (Wang et al., 2019a; Calori et al., 2008) in this systematic review used less conventional orthobiologics in their studies. Calori et al. (Calori et al., 2008) compared PRP with rhBMP-7 on 120 long bone non-unions. Both clinical and radiological union were better with rhBMP-7. So, it could be the object of interest in future studies. The bone marrow stem cell Screen-Enrich-Combine Circulating System (SECCS) Therapy studied by Xin Wang et al. (Wang et al., 2019a) promises good outcomes.

The findings of this systematic review highlight the beneficial role of orthobiologics not only for bone non-unions but also for delayed union. However, it is worth mentioning the difference between bone non-unions and delayed union. Delayed union can be defined as the cessation of the periosteal response before the fracture successfully has been healed. Conversely, nonunion is the cessation of both the periosteal and endosteal healing responses without bridging (Marsh, 1998). This difference may be an important factor when testing the role of orthobiologics. The studies analyzed showed better outcomes and shorter healing time for delayed unions treated with orthobiologics. Healing rate and bone union time seem to be important factors that drive the outcome of treatment of delayed union and non-union. A recent systematic review and meta-analysis confirms that local administration of PRP should be used in cases of delayed union to shorten the treatment period and increase the healing rate (Li et al., 2022). To date most of the studies used platelet derived products confirming the ability of PRP to recruit more progenitor cells and stimulate osteoblast activity to release cytokines in the surrounding environment to improve bone healing. Only one study tested BMAC as orthobiologic for delayed union with good clinical outcomes (Canton et al., 2023). However the nature of the study, without any control group, raises concern about the clinical applicability and efficacy of bone marrow derived products for delayed unions.

Moreover, this systematic review suggests that orthobiologics, particularly PRP, can play a beneficial role in managing delayed and non-union fractures. The studies generally reported no complications related to orthobiologics, indicating that they are a safe option for treating non-union fractures. Orthobiologics can reduce healing time, improve union rates, and enhance functional outcomes in patients with non-union fractures. The choice of orthobiologic may depend on factors such as patient characteristics, fracture type, and surgeon preference, but more evidence is needed.

Future direction could be to set up level 1 studies in order to compare different type of orthobiologics on the same fracture pattern or the same orthobiologic on different fracture pattern. Studies to compare different protocol of orthobiologic preparation are needed also.

As this review adopts a systematic approach, it is important to note that the inherent limitations within the included studies are reflected in our analysis. Various factors contribute to the challenge of directly comparing findings across individual studies, such as the heterogeneity in PRP and other orthobiologics preparation methods, activation processes, variations in bone pathology, anatomical placement, timing of application, and outcome metrics. Incorporating studies that assess radiological evidence of fracture healing introduces an inherent risk of errors and biases in the interpretation and reporting of radiographs. Participant selection bias represents a potential source of distortion in our findings. Another bias is related to the phenomenon in the literature to favor the dissemination of positive results (Joober et al., 2012; Jakobsen et al., 2010). It is crucial to acknowledge the possibility that studies with negative results might exist but remain unreported in the current literature, contributing to a potential underrepresentation of unfavourable outcomes.

## 5 Conclusion

This systematic review provides evidence supporting the use of orthobiologics as an effective and safe option for managing delayed and non-union fractures, with the potential to improve patient outcomes and reduce healing times. However, further RCTs and larger-scale level 1 studies are needed to confirm these findings and establish best practices for the use of orthobiologics in clinical practice.

## Funding/support statement

This research did not receive any specific grant from funding agencies in the public, commercial, or not-for-profit sectors.

## CRediT authorship contribution statement

**Lorenzo Impieri:** Writing – original draft, Visualization, Methodology, Formal analysis, Data curation. **Andrea Pezzi:** Writing – original draft, Visualization, Methodology, Investigation, Formal analysis, Data curation. **Henrique Hadad:** Writing – original draft, Investigation, Formal analysis, Data curation. **Giuseppe M. Peretti:** Writing – review & editing, Methodology, Conceptualization. **Laura Mangiavini:** Writing – review & editing, Writing – original draft, Data curation, Conceptualization. **Nicolò Rossi:** Writing – review & editing, Writing – original draft, Visualization, Validation, Methodology, Formal analysis, Conceptualization.

## Declaration of competing interest

The authors have no conflicts of interest relevant to this article.

## Data Availability

No data was used for the research described in the article.

## References

[bb0005] Acosta-Olivo C., Garza-Borjon A., Simental-Mendia M., Vilchez-Cavazos F., Tamez-Mata Y., Peña-Martinez V. (2017). Delayed union of humeral shaft fractures: comparison of autograft with and without platelet-rich plasma treatment: a randomized, single blinded clinical trial. Arch. Orthop. Trauma Surg..

[bb0010] Bielecki T., Gazdzik T.S., Szczepanski T. (2008). Benefit of percutaneous injection of autologous platelet-leukocyte-rich gel in patients with delayed union and nonunion. Eur. Surg. Res..

[bb0015] Calori G.M., Tagliabue L., Gala L., d’Imporzano M., Peretti G., Albisetti W. (2008). Application of rhBMP-7 and platelet-rich plasma in the treatment of long bone non-unions. Injury.

[bb0020] Canton G., Tomic M., Tosolini L., Di Lenarda L., Murena L. (2023). Use of bone marrow aspirate concentrate (BMAC) in the treatment of delayed unions and nonunions: a single-center case series. Acta Biomedica Atenei Parmensis..

[bb0025] Caplan A.I. (1991). Mesenchymal stem cells. J. Orthop. Res..

[bb0030] Carragee E.J. (1997). The clinical use of magnetic resonance imaging in pyogenic vertebral osteomyelitis: Spine..

[bb0035] Cen C, Cao Y, Zhang Y, et al. Synergistic effects of autologous platelet-rich plasma combined with an extracorporeal shock wave in treatment of long diaphysis aseptic nonunion. Orthopaedics & Traumatology: Surgery & Research. Published online September 2022:103417. doi:10.1016/j.otsr.2022.103417. 37010140

[bb0040] Chiang C.C., Su C.Y., Huang C.K., Chen W.M., Chen T.H., Tzeng Y.H. (2007). Early experience and results of bone graft enriched with autologous platelet gel for recalcitrant nonunions of lower extremity. Journal of Trauma: Injury, Infection & Critical Care..

[bb0045] Costa M.L., Achten J., Knight R. (2020). Effect of incisional negative pressure wound therapy vs standard wound dressing on deep surgical site infection after surgery for lower limb fractures associated with major trauma: the WHIST randomized clinical trial. JAMA.

[bb0050] Dhillon M.S., Patel S. (2022). Why OrthoBiologics?. Journal of Clinical Orthopaedics and Trauma..

[bb0055] Dimitriou R., Mataliotakis G.I., Angoules A.G., Kanakaris N.K., Giannoudis P.V. (2011). Complications following autologous bone graft harvesting from the iliac crest and using the RIA: a systematic review. Injury.

[bb0060] Duramaz A., Ursavaş H.T., Bilgili M.G., Bayrak A., Bayram B., Avkan M.C. (2018). Platelet-rich plasma versus exchange intramedullary nailing in treatment of long bone oligotrophic nonunions. Eur. J. Orthop. Surg. Traumatol..

[bb0065] Elliott D.S., Newman K.J.H., Forward D.P. (2016). A unified theory of bone healing and nonunion: BHN theory. The Bone & Joint Journal..

[bb0070] Emara K.M. (2015). Recent biological trends in management of fracture non-union. WJO.

[bb0075] Fayaz H.C., Giannoudis P.V., Vrahas M.S. (2011). The role of stem cells in fracture healing and nonunion. International Orthopaedics (SICOT)..

[bb0080] Foster T.E., Puskas B.L., Mandelbaum B.R., Gerhardt M.B., Rodeo S.A. (2009). Platelet-rich plasma: from basic science to clinical applications. Am. J. Sports Med..

[bb0085] Galasso O., Mariconda M., Romano G. (2008). Expandable intramedullary nailing and platelet rich plasma to treat long bone non-unions. J. Orthop. Trauma.

[bb0090] Gálvez-Sirvent E., Ibarzábal-Gil A., Rodríguez-Merchán E.C. (2020). Treatment options for aseptic tibial diaphyseal nonunion: a review of selected studies. EFORT Open Reviews..

[bb0095] Giannoudis P.V., Einhorn T.A., Marsh D. (2007). Fracture healing: the diamond concept. Injury.

[bb0100] Gołos J., Waliński T., Piekarczyk P., Kwiatkowski K. (2014). Results of the use of platelet rich plasma in the treatment of delayed union of long bones. Ortop. Traumatol. Rehabil..

[bb0105] Hak D.J., Fitzpatrick D., Bishop J.A. (2014). Delayed union and nonunions: epidemiology, clinical issues, and financial aspects. Injury.

[bb0110] Ho-Shui-Ling A., Bolander J., Rustom L.E., Johnson A.W., Luyten F.P., Picart C. (2018). Bone regeneration strategies: engineered scaffolds, bioactive molecules and stem cells current stage and future perspectives. Biomaterials.

[bb0115] Hurley E.T., Lim Fat D., Moran C.J., Mullett H. (2019). The efficacy of platelet-rich plasma and platelet-rich fibrin in arthroscopic rotator cuff repair: a meta-analysis of randomized controlled trials. Am. J. Sports Med..

[bb0120] Jakobsen A.K., Christensen R., Persson R., Bartels E.M., Kristensen L.E. (2010). And now, e-publication bias. BMJ.

[bb0125] Jamal M.S., Hurley E.T., Asad H., Asad A., Taneja T. (2022). The role of platelet rich plasma and other orthobiologics in bone healing and fracture management: a systematic review. Journal of Clinical Orthopaedics and Trauma..

[bb0130] Jones I.A., Togashi R., Wilson M.L., Heckmann N., Vangsness C.T. (2019). Intra-articular treatment options for knee osteoarthritis. Nat. Rev. Rheumatol..

[bb0135] Joober R., Schmitz N., Annable L., Boksa P. (2012). Publication bias: what are the challenges and can they be overcome?. J. Psychiatry Neurosci..

[bb0140] Kanakaris N.K., Giannoudis P.V. (2007). The health economics of the treatment of long-bone non-unions. Injury.

[bb0145] Kolade O, Patel K, Ihejirika R, et al. Response to Amin et al regarding: “Efficacy of liposomal bupivacaine in shoulder surgery: a systematic review and meta-analysis.” J. Shoulder Elb. Surg. 2020;29(5):e213-e214. doi:10.1016/j.jse.2020.01.001. 32305111

[bb0150] Li Q., Song J., Li X. (2020). Differentiation of intraspinal tuberculosis and metastatic cancer using magnetic resonance imaging. IDR.

[bb0155] Li S., Xing F., Luo R., Liu M. (2022). Clinical effectiveness of platelet-rich plasma for long-bone delayed union and nonunion: a systematic review and meta-analysis. Front Med (Lausanne)..

[bb0160] Malhotra R., Kumar V., Garg B. (2015). Role of autologous platelet-rich plasma in treatment of long-bone nonunions: a prospective study. Musculoskelet. Surg..

[bb0165] Mariconda M., Cozzolino F., Cozzolino A., D’Agostino E., Bove A., Milano C. (2008). Platelet gel supplementation in long bone nonunions treated by external fixation. J. Orthop. Trauma.

[bb0170] Marsh D. (1998). Concepts of fracture union, delayed union, and nonunion. Clin. Orthop. Relat. Res..

[bb0175] Marx R.E. (2001). Platelet-rich plasma (PRP): what is PRP and what is not PRP?. Implant. Dent..

[bb0180] Miller S.A., Forrest J.L. (2001). Enhancing your practice through evidence-based decision making: PICO, learning how to ask good questions. Journal of Evidence Based Dental Practice..

[bb0185] Nauth A., Lee M., Gardner M.J. (2018). Principles of nonunion management: state of the art. J. Orthop. Trauma.

[bb0190] Patel S., Dhillon M.S., Aggarwal S., Marwaha N., Jain A. (2013). Treatment with platelet-rich plasma is more effective than placebo for knee osteoarthritis: a prospective, double-blind. randomized trial. Am J Sports Med..

[bb0195] Ranjan R., Kumar R., Jeyaraman M., Arora A., Kumar S., Nallakumarasamy A. (2023). Autologous platelet-rich plasma in the delayed union of long bone fractures – a quasi experimental study. J. Orthop..

[bb0200] Rodriguez-Merchan E.C., Moreno-Garcia A. (2021). Orthobiologics: current role in orthopedic surgery and traumatology. ABJS.

[bb0205] Rossi N., Hadad H., Bejar-Chapa M. (2023). Bone marrow stem cells with tissue-engineered scaffolds for large bone segmental defects: a systematic review. Tissue Eng. Part B Rev..

[bb0210] Rossi N., Sciancalepore F., Daolio P.A., Verdoni F., Mangiavini L. (2023). Huntington procedure for the treatment of tibial nonunion in a 17-years old male: a case report. Int. J. Surg. Case Rep..

[bb0215] Samuel G., Menon J., Thimmaiah S., Behera G. (2018). Role of isolated percutaneous autologous platelet concentrate in delayed union of long bones. Eur. J. Orthop. Surg. Traumatol..

[bb0220] Sanchez M., Anitua E., Cugat R. (2009). Nonunions treated with autologous preparation rich in growth factors. J. Orthop. Trauma.

[bb0225] Say F., Türkeli E., Bülbül M. (2014). Is platelet-rich plasma injection an effective choice in cases of non-union?. Acta Chir. Orthop. Traumatol. Cechoslov..

[bb0230] Singh A.K., Shetty S., Saraswathy J.J., Sinha A. (2013). Percutaneous autologous bone marrow injections for delayed or non-union of bones. J. Orthop. Surg. (Hong Kong).

[bb0235] Tarallo L., Mugnai R., Adani R., Catani F. (2012). Treatment of the ulna non-unions using dynamic compression plate fixation, iliac bone grafting and autologous platelet concentrate. Eur. J. Orthop. Surg. Traumatol..

[bb0240] Tay W.H., De Steiger R., Richardson M., Gruen R., Balogh Z.J. (2014). Health outcomes of delayed union and nonunion of femoral and tibial shaft fractures. Injury.

[bb0245] Ulivi M., Meroni V., Viganò M. (2023). Micro-fragmented adipose tissue (mFAT) associated with arthroscopic debridement provides functional improvement in knee osteoarthritis: a randomized controlled trial. Knee Surg. Sports Traumatol. Arthrosc..

[bb0250] Urist M.R. (1965). Bone: formation by autoinduction. Science.

[bb0255] Wang X., Chu W., Zhuang Y. (2019). Bone mesenchymal stem cell-enriched β-tricalcium phosphate scaffold processed by the screen-enrich-combine circulating system promotes regeneration of diaphyseal bone non-union. Cell Transplant..

[bb0260] Wang X., Chen Y., Liu Y. (2019). Reporting items for systematic reviews and meta-analyses of acupuncture: the PRISMA for acupuncture checklist. BMC Complement. Altern. Med..

